# Mismatch Between Physical Health and Job Demands among Older Workers by Full Retirement Age

**DOI:** 10.1093/geroni/igab046.3286

**Published:** 2021-12-17

**Authors:** Theresa Andrasfay

**Affiliations:** University of Southern California, Los Angeles, California, United States

## Abstract

Gradual increases to the Social Security full retirement age (FRA) from 65 to 67 were justified by improvements in the health of the older population and a general shift toward less physically demanding jobs. These two trends have been studied independently, but it is important to consider the agreement of these two factors—job demands and health—to understand whether those expected to work longer to receive full benefits have compatible health and job characteristics to do so. Using data from the 1992-2018 waves of the Health and Retirement Study, I observe 19,383 working individuals with FRA ranging from 65-67 while they are approaching retirement (ages 51-60). I compare the prevalence of person-work mismatch—defined by the co-occurrence of physical health conditions and self-reported physical job demands—by FRA. I find that individuals with an older FRA are less likely to be employed in physically demanding jobs while having arthritis. However, they are more likely to be employed in physically demanding jobs while having pain or fair/poor self-assessed health and are more likely to be employed in jobs requiring frequent stooping, kneeling, or crouching while simultaneously having difficulty with these activities. The co-occurrence of physically demanding work while having multiple mobility limitations has remained stable across the FRA cohorts. These findings indicate that older workers expected to work longer to receive full benefits have not experienced substantial improvements in the compatibility between their physical health and job demands that would facilitate working longer, and by some measures compatibility has declined.

